# Pien Tze Huang (PZH) as a Multifunction Medicinal Agent in Traditional Chinese Medicine (TCM): a review on cellular, molecular and physiological mechanisms

**DOI:** 10.1186/s12935-021-01785-3

**Published:** 2021-03-03

**Authors:** Zhiliang Chen

**Affiliations:** Fujian Provincial Key Laboratory of PTH Natural Medicine Research and Development, Zhangzhou PTH Pharmaceutical CO., LTD, Zhangzhou, 363000 China

**Keywords:** Pien tze huang (PZH), Traditional chinese medicine (TCM), Medicinal treatment, Mechanism of sction, Physiology, Cellular effects, Molecular

## Abstract

**Relevance:**

Pien Tze Huang (PZH) is a well-known Traditional Chinese Medicine (TCM), characterized by a multitude of pharmacological effects, such as hepatoprotection and inhibition of inflammation and cell proliferative conditions. Many of these effects have been validated at the cellular, molecular and physiological levels but, to date, most of these findings have not been comprehensively disclosed.

**Objectives:**

This review aims to provide a critical summary of recent studies focusing on PZH and its multiple pharmacological effects. As a result, we further discuss some novel perspectives related to PZH’s mechanisms of action and a holistic view of its therapeutic activities.

**Methods:**

A systematic review was performed focusing on PZH studies originated from original scientific resources. The scientific literature retrieved for this work was obtained from International repositories including NCBI/PubMed, Web of Science, Science Direct and China National Knowledge Infrastructure (CNKI) databases.

**Results:**

The major active componentes and their potential functions, including hepatoprotective and neuroprotective effects, as well as anti-cancer and anti-inflammatory activities, were summarized and categorized accordingly. As indicated, most of the pharmacological effects were validated in vitro and in vivo*.* The identification of complex bioactive components in PZH may provide the basis for further therapeutic initiatives.

**Conclusion:**

Here we have collectively discussed the recent evidences covering most, if not all, pharmacological effects driven by PZH. This review provides novel perspectives on understanding the modes of action and the holistic view of TCM. The rational development of future clinical trials will certainly provide evidence-based medical evidences that will also confirm the therapeutic advantages of PZH, based on the current information available.

## Background

Natural mediciations have been used in different world regions for thousands of years. They are currently used as alternative or adjunstive therapeutic strategies for diferent disorders [1–8]. Traditional Chinese Medicine (TCM) as one of most established medical systems worldwide has been recognized by the World Health Organization by including TCM diagnostic patterns into the new revision of the International Classification of Diseases code [9–14]. TCM has drawn significant research attention in the western world. Pien Tze Huang (PZH) is a well-known TCM formula used to treat liver diseases, pro-inflammatory conditions and cancer in China and other Asian countries for hundreds of years [15, 16]. PZH is mainly composed of musk, Calculus bovis (Niuhuang or ox's gallstone), snake gall (Shedan) and *Panax notoginseng* (Sanqi) roots [1]. These natural constituents can provide heat-clearing (antipyretic), anti-inflammatory and detoxification effects. Nowadays, PZH is listed as one of the national class-1 protected TCMs.

Despite its long time history and popularity, PZH has only recently been documented in the Western literature. With the increasing amount of studies conducting on the PZH’s pharmacological effects, deciphering its mechanism of action (MOA), as well as characterizing all related bioactive compounds, has been of significant priority. Therefore, this study was aimed to comprehensively review the pharmacological effecs and clinical applications of PZH for different disorders. This study discusses the chemical compositions, active components and hepatoprotective, neuroprotective, anti-cancer and anti-inflammatory functions of PZH on cellular, molecular and physiological levels. We further discuss some novel perspectives related to PZH’s MOA and a holistic view of its therapeutic activities.

We did a systematic literature review by initially searching all the related scientific publications in the NCBI/PubMed, Web of Science, Science Direct and CNKI databases. The most relevant literature was selected for further discussion in this review. We sumarize the findings of the reviewed articles by categorizing them into specific topics, according to PZH’s major active componentes and their biological effects. To the best of our knowledge, this study would be one of the pioneering articles focusing on PZH in the western literature. We expect the findings of this study can contribute novel perspectives on better understanding of the MOA and the holistic view underlying the TCM PZH.

## Major active components

### Panax notoginseng roots

PZH is composed of four TCM ingredients, including Radix et Rhizoma Notoginseng (85%), Moschus (3%), Calculus Bovis (5%), and Snake Gall (7%) [17–19]. *Panax notoginseng (P. notoginseng),* also known as Chinese ginseng, is distributed primarily in southwest China, mainly due to its peculiar growth habitat. This plant contains various natural compounds, including saponins, flavonoid glycosides, amino acids and other active chemicals [20]. Among these, a distinct type of amino acid, known as Beta-N-oxalyl-l-alpha, beta-diaminopropionic acid (Beta-ODAP), functions as a hemostatic agent [21]. It has been reported that dencichine is able to shorten bleeding time, lower blood pressure and even decrease the heart rate [22].

Compounds derived from *P. notoginseng* have shown different pharmacological functions in a variety of physiological conditions. Certain saponins (i.e. panaxatriols) have been used for treatment of arrhythmia induced by various drugs. Other bioactive constituents, such as trilinolein, have been reportedly capable of reducing myocardial oxygen consumption and oxygen utilization rate as well as of improving the cerebrovascular flow [23–25]. *P. notoginseng* extract can also improve humoral immune function and act as putative anti-tumor agent [26]. Furthermore, other pharmacological activities, such as analgesic, anti-inflammatory and anti-aging effects, have been reported [27].

### Snake gallbladder

Snake gallbladder stores bile, a digestive fluid, which is originally produced by the liver. Therefore, snake gallbladder is known to contain a number of bile salts and acids, such as taurocholic acid (produced by conjugation of cholic acid and taurine), tauroursodeoxycholic acid, taurodeoxycholic acid and free cholic acid, as well as cholesterol [28]. Among these constituents, taurocholic acid is one of the most abundant components from the snake gallbladder.

A number of studies have shown that snake gallbladder possesses a variety of activities, including anti-inflammatory, anti-asthmatic, antitussive, expectorant and bacteriostatic. Interestingly, several studies have demonstrated the immune regulatory as well as blood pressure lowering effects of gallbladder in different animal and human studies [28–32].

### Calculus Bovis

Calculus Bovis are dried gallstones of cattle and/or buffalo, typically used in Chinese herbology. Some of them are stones (bezoars) derived from bile or hepatic ducts. Studies have shown that bezoars may contain cholic and deoxycholic acids, cholesterol, bilirubin, ergosterol, vitamin D and minerals, including sodium, calcium, magnesium, zinc and others [33]. Other components have also been identified, including carotenoids, alanine, glycine and other amino acids, as well as mucin, fatty acids and certain peptides [34]. Some reports have found that bezoar may contain sedative, anticonvulsant and/or antipyretic activities [35]. Bilirubin is a byproduct of red blood cell breakage, which is also one of the main components of Calculus bovis. Interestingly, supplemental bilirubin has been shown to lower blood pressure and reduce heart rate [36].

The aqueous component of Calculus bovis, which contains cholic acids such as deoxycholic acid, can induce gallbladder contraction, soften the sphincter of biliary duct (i.e. sphincter of Oddi) and promote bile secretion. Therefore, this component clearly shows beneficial effects on biliary disorders [37]. The acidic components of Calculus bovis have reportedly exhibited a protective effect against acute and chronic liver damage, caused by carbon tetrachloride in rats [38]. For instance, intravenous drip of Benzoic acid, can significantly increase red blood cells in rabbits [39]. In summary, bezoars have shown to provide anti-inflammatory, hemostatic and lipid-lowering effects into formulations[6].

### Musk

Musk is a dried product of glandular secretions of mature male musk deer, such as *Moschus berezovskii*, *M. sifanicus* and *M. moschiferus* [40]. Studies have shown that musk contains macrocyclic compounds (such as muscone), steroids (such as testosterone), estradiol, cholesterol, amino acids (such as aspartic acid), inorganic salts and others [41]. Musk has bi-directional effects on the central nervous system (CNS), exhibiting neuronal excitation at low doses and inhibition at high doses [42, 43]. Muscone, one of the main components of Musk, has also been shown to enhance the ability of the CNS to resist hypoxia and thus, to improve cerebral circulation [44]. Musk can also stimulate cardiac function, by increasing the amplitude of cardiac contraction and enhancing myocardial function [44]. As such, it has been used as a preventive and therapeutic agent on ischemic heart disorders caused by thrombosis [45]. Musk extracts display anti-inflammatory effects which are similar to those provided by hydrocortisone [46, 47]. Moreover, administration of musk extracts can stimulate the uterus and enhance uterine contraction [44]. At high concentrations, it has been reported that musk extracts can also inhibit proliferation of certain human cancers in vitro, including Ehrlich ascites cancer cells and sarcoma S180 cells [48].

## Chemical characterization of PZH

Due to their biological effects, extensive effort has been dedicated to isolate and characterize, in more details, the chemical constituents of the major components of PZH.

Five active ingredients of *P. notoginseng*, including saponin R1, ginsenoside Rg1, ginsenoside Rb1, thymol and sodium taurocholate, have been identified and separated by Mixed Micellar Capillary Electrokinetic Chromatography (Mixture Ms-MEKC) [49]. Other four main components, including thymol, have been determined by high performance liquid chromatography (HPLC) [50]. An efficient method using ultra-performance LC coupled with triple quadrupole MS (UPLC-QQQ-MS) has been developed for the rapid determination of 12 main compounds (notoginsenoside R1, ginsenoside Rb1, ginsenoside Rg1, ginsenoside Rg3, cholic acid, deoxycholic acid, hyodeoxycholic acid, ursodesoxycholic acid, chenodeoxycholic acid, sodium taurochenodeoxycholate, sodium tauroursodeoxycholate, muscone) in PZH [51]. More recently, a total of nine saponins, eleven bile acids, taurine and muscone have been characterized as anti-inflammatory constituents in PZH [52]. These bioactive compounds have been identified as potent inhibitors of TNF production, with IC50 values ranging between 12 to 147 μM in vitro [52].

An optimized methodology, based on ultra-performance liquid chromatography with triple quadrupole mass spectrometry (UPLC–MS/MS), has been developed for the rapid quantification and pharmacokinetic analysis of six particular components (i.e. notoginsenoside R1, ginsenosides Re, Rg1, Rb1, Rd, and muscone) [18]. These natural compounds could be identified and extracted from rat plasma, after oral administration of PZH [18]. Among these identified constituents, saponins and ginsenosides have been shown to protect against fibrosis, superoxide formation, and to lower the levels of serum triglycerides [53, 54]. It has been proposed that compounds isolated from both musk and notoginseng roots mainly function to modulate cell death processes and vascular spasms, while those isolated from Calculus bovis and snake gallbladder mostly serve as anti-inflammatory agents [23, 53, 54]. The detailed chemical composition of all constituents serves as a basis for proper characterization of specific components that provide liver protection and lipid lowering functions, as well as anti-inflammatory, anti-oxidation and/or anti-fibrosis activities. To further understand the pharmacological actions of PZH, it is important to verify potential synergistic effects among the active chemicals. The metabolic outcomes of these interactions could be primarily analyzed by integrated PK/PD-metabolomics (Table 1).

**Table 1:** Active chemicals identified from the four major component of Pien Tze Huang (PZH). Their putative biological functions and respective references are also listed

Components	Active chemicals identified	Biological functions	References
Musk	Macrocyclic compounds such as musk ketone, steroids such as testosterone, estradiol, cholesterol, a variety of amino acids such as aspartic acid, inorganic salts and others	Resist hypoxia and improve cerebral circulation, stimulate cardiac function, increase the amplitude of cardiac contraction and enhance myocardial function, anti-inflammatory effect, stimulate uterus and enhance uterine contraction	[20, 24–27, 29, 37]
Calculus bovis	Cholic acid, deoxycholic acid, cholesterol, bilirubin, ergosterol, vitamin D, sodium, calcium, magnesium, zinc, carotenoids, alanine, glycine and other amino acids, as well as mucin, fatty acids and peptides	Sedative, anticonvulsant and antipyretic effects, lower blood pressure and reduce heart rate, induce gallbladder contraction, relax the sphincter of biliary duct and promote bile secretion, anti-inflammatory, hemostatic and lipid-lowering effects, hepatoprotective effects	[14, 15, 17–20]
Snake gall	Taurocholic acid, taurogoosedeoxycholic acid, taurodeoxycholic acid, free cholic acid and cholesterol	Anti-inflammatory, antitussive, bacteriostatic, expectorant, antiasthmatic effects and used in the immune regulation and lowering blood pressure	[12, 13]
The root of Panax notoginseng	Saponins, flavonoid glycosides, amino acids and other active chemicals	Hemostatic effect; shorten bleeding time, lower blood pressure and slow heart rate; reduce myocardial oxygen consumption and oxygen utilization rate, expand cerebrovascular, and enhance cerebrovascular flow; improve humoral immune function; analgesic, anti-inflammatory and anti-aging activities	[4–11]
Pien Tze Huang	Notoginsenoside R1, ginsenosides Re, ginsenosides Rg1, ginsenoside Rb1, ginsenosides Rd, muscone, bile acids, thymol, sodium taurocholate, taurine	Protect against fibrosis, superoxide formation, and lower serum triglyceride; protect from cell death and antivascular spasm; anti-inflammatory effects	[7, 20, 31–36]

## Relevant biological functions of PZH

### Hepatoprotective effects

#### Molecular mechanisms of PZH-mediated hepatoprotection

**Fig. 1:**
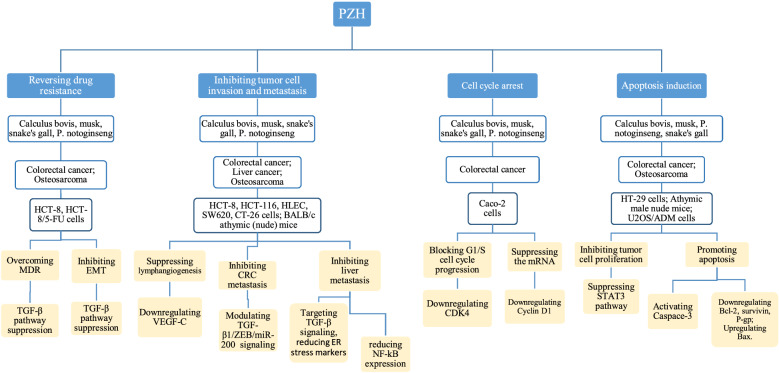
The effects of PZH extracted medications on different cells, animal and human models of different cancers and the proposed mechanisms of actions

A variety of in vivo studies have consistently shown that PZH can significantly lower serum ALT and AST levels and, concomitantly, modulate inflammatory cell infiltration and other pathological changes [55, 56]. The hepatoprotective effects of PZH were first reported in the Western literature in 2004 [57]. Using a carbon tetrachloride (CCl4)-induced animal model to evaluate drug-dependent hepatoprotective effects, PZH has shown a positive outcome against liver damage [55]. Although no significant effects on cell proliferation were observed, PZH was able to regulate a series of responsive elements, mediated by AP-1, CRE and NF-κB, in hepatoma cells [55]. This hepatoprotective effect was not altered even after the substitution for natural musk (nPZH) with a formulated PZH substitute (sPZH) [57]. Both nPZH and sPZH were able to protect the liver, to a similar extent, against CCl4- and galactosamine-induced hepatotoxicity, while sharing a similar patterns of regulation towards those responsive DNA elements [57]. Chromatographic analyses of nPZH and sPZH also revealed that their chemical components were very similar [57]. Recently, hepatoprotective effects were also identified in a TCM variant called PZH Ganbao (PZH combined with other hepatoprotective components) [58]. Using a high fat diet (HFD)-induced non-alcoholic fatty liver disease (NAFLD) model, PZH could significantly (i) revert the hepatic lipid degeneration, (ii) reduce the levels of AST, ALT, gamma-GT and TG, (iii) improve hepatocyte steatosis, (iv) reduce hepatocyte necrosis and inflammatory cell infiltration, and (v) regulate the expression of key factors of lipid metabolism (Farnesoid X Receptor (FXR), Small Heterodimer Partner (SHP), Sterol-regulatory Element Binding Protein-1c (SREBP-1c)) [58]. These PZH-mediated effects suggest a highly regulated mechanism towards lipid metabolism. Moreover, it also provides a new way of exploring the prevention and treatment of NAFLD. Other studies have also shown that PZH can significantly inhibit the expression of key genes involved in the metabolism of glycolipids, in acute or chronic alcoholic liver injury models, including PPAR-gamma, SREBP2, HMGCR, IL-β, and MCP-1 [59]. In addition, PZH can exert a hepatoprotective effect in an alcoholic and HFD in vivo model [56]. In this model, PZH has ameliorated the hepatic function/pathology and the impairment in lipid metabolism, suggesting not only a regulatory function in lipid metabolism but also a putative role in alcohol detoxification [56]. Actually, PZH appears to promote the regulation of FXR-SHP-SREBP1c signaling, a key pathway in lipid metabolism, in a HFD model [60]. Figure 1 shows a summary on the effects of different medications extracted from PZH on cells, animal and human models of different malignancies and also the involved MOAs.

Clinical evidences are required to confirm whether PZH could be used as a hepatoprotective agent on the treatment of alcoholic liver disease (ALD) with obesity. However, PZH can significantly reduce serum homocysteine (Hcy) level and inhibit the PERK/eIF2ɑ pathway, suggesting a hepatoprotective effect through the modulation of Hyc-induced ER stress [56]. So far, it has been shown that high levels of Hcy affect lipid metabolism in the liver and also induce ER stress. This explains, to some extent, how PZH treatment may avoid liver damage. Further analyses of PZH’s hepatoprotective activities, based on bile duct ligation and acute alcohol intoxication in vivo models, also revealed that PZH can (i) decrease necrotic and apoptotic cell rates, (ii) downregulate transaminase levels, (iii) diminish the extent of fibrosis, (iv) down-regulate the levels of TGF-β producing cells in the liver and, ultimately, (v) enhance cell proliferation in the liver [1]. TGF-β is a critical regulator of several chronic liver diseases, including liver fibrosis. Altogether, these roles indicate that the underlying MOA of PZH may be related to the modulation of TGF-β expression in the liver. These studies have indicated that PZH may have multiple pharmacological effects, which can be useful for the treatment of liver diseases including NAFLD. Nevertheless, identifying the complete molecular pathways that link the hepatoprotective networks elicited by PZH needs further investigation.

#### Hepatoprotective effects through regulation of ER stress and autophagic pathways

Endoplasmic reticulum (ER) is a critical organelle, present in all eukaryotic cells, which is mainly responsible for (i) protein synthesis, folding and transport, (ii) sequestration of calcium and (iii) production and storage of glycogen, steroids, and other macromolecules [61]. Endoplasmic reticulum stress (ERS) is a cellular condition derived from the accumulation of unfolded proteins in the ER. The rough ER (RER) is composed by ER dotted with ribosomes, and it is typically abundant in hepatic cells. RER is essential for the proper folding of secretory proteins, caciulm storage, and lipid and cholesterol synthesis. Therefore, ER in the hepatic cells could be eventually susceptible to an overload of misfolded proteins, leading to ERS.

PZH has been widely used in the treatment of various types of liver diseases [1, 55, 56, 60]. It has been reported to ameliorate liver injury in vivo, after administration of alcohol and high-fat diet in rat models [56]. It can reduce serum levels of ALT, AST, ALB, TBIL, TG and TCH, which are liver damage markers widely used in the clinic. It also maintains liver structure and protects hepatocyte morphology. Moreover, it reduces the degree of apoptosis and necrosis. It also reduces inflammatory cell infiltration and improves steatosis. The reduction of elevated Hcy levels, and the expression of GRP78, GRP94, p-eIF2a, Caspase-9 and Caspase-12can provide an explanation for those effects [56]. It is suggested that PZH may play a hepatoprotective role via regulating Hcy-induced ERS mediated by PERK/eIF-2alpha signaling pathway.

Studies have shown that saponins isolated from *P. notoginseng*, can down-regulate ethanol-mediated oxidative stress to decrease liver injury [62]. Interestingly, these saponins can also modulate palmitate-triggered ER stress, leading to potential neuroprotective effects in vitro and in vivo [63, 64]. Consistently, total saponins of P. notoginseng can significantly reduce the expression of ER stress markers, such as GRP78, p-eIF2a, p-PERK, as well as inflammation-related factors, such as phosphorylated NF-kB, TNF-alpha and IL-1β in aging rats [65], suggesting novel pharmacological routes, and thus potential drug targets, prone to treatment (Fig. 1).

Autophagy is a lysosome-dependent process of cell self-degradation, largely conserved in eukaryotes. This process is highly regulated to disassemble unnecessary or dysfunctional components, and to allow the orderly degradation and recycling of cellular components [66]. It has been shown that autophagy not only plays an important role in normal physiological processes, but also impacts the progression of many diseases. Under physiological conditions, autophagy is maintained at relatively low levels, and in a controllable homeostatic state. One of the main functions of autophagy is to promote cell survival [67]. In certain pathological conditions, autophagy is reportedly able to eventually lead to cell death. For instance, oxidative stress can promote autophagic cell death and, eventually, drive tissue deterioration in some liver diseases.

Autophagy also plays an important role in the regulation of inflammatory processes. Recent studies have shown that autophagy signaling cascades and/or autophagy-related proteins may coordinate the balance between inflammatory response and inflammasomes [68]. Inflammasomes are large intracellular multiprotein complexes and play a critical role in bacterial infection, metabolism regulation, and innate immunity [69]. For instance, in autophagy-related (Atg) gene deficient cells, lipopolysaccharide (LPS)-induced activation of NF-kB largely increases the expression of inflammatory cytokines, which can lead to the aggravation of inflammatory responses. LPS can also activate TLR4 in both *Atg16L1* and *Atg7*-deficient mouse macrophages; thus, increasing the expression of genes related to inflammatory corpuscle and cytokines such as IL-1β and IL-18. Therefore, attenuating autophagy may promote inflammation, while promoting autophagy may interrupt an inflammatory response.

Some studies have shown that PZH might play additional roles in regulating autophagy. In the cholecystitis model, the expression of the autophagy marker LC3 is significantly decreased while, after PZH treatment, LC3 expression is significantly increased, suggesting that the protective effect of PZH on cholecystitis may be mediated by autophagy. However, further studies are needed to determine the specific molecular mechanism still requires further exploration.

The PI3K/Akt/mTOR signaling pathway is closely related to autophagy, and it plays an important role in regulating apoptosis and cell proliferation [15]. Actually, a decrease on PI3K/Akt/mTOR activity can induce apoptosis in human osteosarcoma (OS) cells (U2OS), and also inhibit the proliferation of drug-resistant OS (MG63/ADM) and ovarian cancer cells [70, 71]. More recently published studies have demonstrated correlation between PI3K/Akt/mTOR activity and occurrence and development of NAFLD and liver fibrosis [72]. Therefore, it is possible that PI3K/Akt/mTOR pathway may play a critical role in the development of NAFLD and liver fibrosis. Considering the inhibitory effect of PZH on PI3K/Akt/mTOR signaling pathway [71], the regulatory effect of this pathway on autophagy and the crucial role of autophagy in NAFLD development, we speculate that PZH may play a therapeutic role by regulating the autophagy-related signaling pathway. In this context, studies have shown that saponins, ginsenosides Rh2 and other active ingredients in P. notoginseng can promote autophagy in a variety of human cells [73]. These results reiterate the potential role of PZH on autophagy, and also infer a therapeutic role by regulating ER stress and autophagic pathways.

### Neuroprotective effects

PZH has been reported to exert beneficial effects in the treatment of several CNS diseases, including multiple sclerosis (MS), stroke, and neuroblastoma [74–78]. Several chemicals with putative anti-inflammatory activity in CNS disorders have been identified in raw PZH extracts [52]. However, more pharmacological studies are needed to validate the therapeutic outcomes of PZH for each neurological condition.

#### Multiple sclerosis

Current evidence of translational and clinical studies have demonstrated that PZH possesses neuroprotective, immuno-regulatory, and anti-inflammatory effects [74–78]. Several chemicals isolated from raw PZH potentially drive anti-inflammatory activities in CNS disorders [52]. However, more pharmacological studies are still needed to validate the possible use of PZH in particular neurological conditions.

Multiple sclerosis (MS) is a chronic inflammatory demyelinating disease of the CNS. The major pathological features of MS involve inflammation, demyelination, and axonal damage. Demyelination is a condition that results from damage of the insulating cover (myelin sheath) of nerve cells, reducing the conduction speed of neural signals in the affected region (i.e. brain and/or spinal cord). Ultimately, it can lead to a range of symptoms, including physical, mental, and/or psychiatric complications.

The exact etiologies and pathogenesis of MS are not completely understood. It has been suggested that etiology of MS is a complex of genetics, viral infection, autoimmune and environmental factors that lead to the destruction of the immune system and/or failure of the myelin-producing cells, which then translate into this complex neurological disorder [79].

There is no definitive cure commercially available for MS. The main medicines used in the treatment of MS are chemosynthetic drugs, including immunosuppressive agents and immunomodulatory agents, which can reduce the recurrence rate and slow down the inflammatory process. However, these non-curative treatments are usually costly and accompanied by different side effects. Therefore, development of more safe, effective and affordable therapeutic drugs is warranted.

Experimental autoimmune encephalomyelitis (EAE) is an experimental animal model for the study of MS [80]. This model has been recognized as an ideal animal model for studying the pathogenesis, treatment and drug development in MS [80].

As previously stated, PZH may offer a multitude of pharmacological activities, such as analgesia, neuroprotection, anti-inflammation and immune regulation [15, 16]. Particularly, it has been employed in the treatment of various disorders such as cancer, liver and cerebrovascular diseases [16, 56]. Therefore, a more detailed analysis of its potential application in MS treatment can be of great value.

The efficacy of PZH for the treatment of MS and other autoimmune diseases has only recently been reported. However, it is not difficult to consider that the pharmacological characteristics of its main ingredients, such as bezoar, P. notoginseng, snake gall and natural musk, could be beneficial for MS treatment. Ginsenosides Rg1 and Rd monomers, derived from extracts of P. notoginseng, are main components that have shown therapeutic effects on the EAE mouse model [81]. These components appear to slow down the severity of EAE disease by improving the degree of inflammatory infiltration and demyelination of spinal cord in vivo. These particular ginsenosides may play a therapeutic role by inhibiting interferon-γ/signal transducer and activator of transcription 3 (STAT3) pathway. Interferon-γ is a cytokine that plays an important role in the control of tumor development. Interferon-γ can affect tumor development by directly regulating the expression of genes related to cell proliferation and apoptosis, such as STAT1, which is a downstream signal protein of interferon-γ signaling pathway. These observations provide additional molecular basis for the pharmacodynamics of PZH [81].

The therapeutic effects of PZH on MS have been initially tested in vivo, using acute EAE rat and relapse-remission EAE mouse models [75]. PZH has been reported to ameliorate the clinical severity of EAE rats. Moreover, PZH is able to improve the clinical symptoms of EAE mice by reducing inflammatory cell infiltration and myelin damage in the CNS [74]. At the molecular level, PZH can reduce inflammation and regulate pro-inflammatory T helper 1 (Th1) and T helper 17 (Th17) cells, since it down-regulates the levels of RORγt, T-bet, interferon-γ, interleukin 17A phosphorylated STAT3 and NF- kB, and also reduces the percentage of Th1 and Th17 cells [74]. Moreover, PZH can remarkably decrease the levels of pro-inflammatory cytokines, such as IL-17A, IL-23, CCL3 and CCL5, and activated transcription factors (p-P65 and p-STAT3) in the CNS, while significantly improving the expression of MBP and Olig2, two important glial markers, without significant toxicity [74]. Altogether, these data strongly suggest a potential clinical use of PZH in the treatment of MS.

#### Stroke

Stroke is a medical condition caused by blood and/or oxygen deprivation in the brain. The high rates of paralysis, recurrence and fatality due to stroke not only seriously endanger the life and health of affected patients, but also impose significant medical and economic burden to their families and the society. In China, cerebrovascular disease has become a leading cause of disability and death in both urban and rural populations, as the incidence rate has increased on a yearly basis. About 2 million new cases of stroke have been diagnosed in China every year, resulting in alarming rates of ~ 1.5 million deaths per year. The incidence of ischemic stroke accounts for 60–80% of all cerebrovascular diseases in China. Therefore, it is of seminal importance to explore the pathogenesis of ischemic stroke as the basis for the further development of safe and effective drugs for clinical use.

Several studies have focused on the therapeutic effects of PZH in cerebral infarction. Certain components of natural PZH have been reportedly effective in the treatment of correlated conditions, including apoplexy. Recently, it has been reported that PZH might function in the brain by inhibiting inflammation after cerebral infarction [77, 82]. These studies have provided a theoretical basis for understanding the pharmacodynamics and biological mechanisms of TCM in the treatment of cerebrovascular disease.

It has been reported PZH can also potentially prevent brain cells from ischemia-induced apoptosis and, consequently, decrease cell death in the hippocampus and cerebellum [76, 83]. Specifically, PZH can reduce cerebral infarct volume, improve neurological deficit, attenuate inflammatory response, and also inhibit neuronal apoptosis in acute ischemic stroke rats [76]. Different MOAs have been attributed to these effects including cell death prevention from apoptosis and/or ROS-dependent oxidative damage in the mitochondria [76]. In this regard, recent studies have shown that PZH can decrease the levels of cytosolic cytochrome C, BCL2-associated X (Bax), p53, cleaved caspase-3, and cleaved caspase-9 and, in contrast, elevate the levels of mitochondrial cytochrome C, B-cell leukemia/lymphoma 2 xL (Bcl-xL), and phosphorylated Akt and GSK-3β, suggesting that the inhibition of mitochondria-mediated neuronal apoptosis as well as attenuation of inflammatory responses could act as therapeutic mechanisms of PZH in ischemic stroke [77].

Interestingly, some studies have suggested that PZH might be able to induce different and even apposite effects on the normal versus cancerous neural cells. In this regard, while PZH could prevent brain cells from apoptosis, it can also induce cancer cell death. For instance, in vitro studies have demonstrated anti-cancer activities of PZH on neuroblastoma cells (SH-SY5Y), suggesting a distinctive effect of PZH in normal versus neural cancer cells [78].

### Anti-cancer effects

#### Colorectal cancer

Colorectal cancer (CRC) is one of the most common malignant tumors of digestive system. CRC is currently the third ranked cancer worldwide in terms of mortality with 22.7 and 15.9 deaths per 100,000 individuals for males and females, respectively worldwide [84].

Chemotherapy is one of the main treatment options for CRC, and different combinations of 5-fluorouracil (5-FU) based drugs are currently prescribed as the standard CRC treatment. In this regard, the efficacy of these chemotherapeutic drugs is not ideal due to significant toxicity and side effects, as well as the putative induction of multi-drug resistance. Although complementary modalities such electroporation and nanoparticles have dramatically improved the therapeutic efficacy of chemotherapeutic agents in CRC, the main limitation of chemotherapy technique is severe cytotoxicity [85–89]. Actually, about 50% of patients with metastatic CRC show a progressive disease within 7–9 months after drug treatment, with a median survival of 20 months and a 5-year survival rate of less than 5%.

At the cellular level, abnormal proliferation and inhibition of apoptosis are hallmarks of tumor progression. Overexpression of the anti-apoptotic genes like *BCL-2* and *BCL-XL* is a classical molecular feature of tumorigenesis and drug resistance. The sensitivity of cells to apoptotic stimuli depends on the antagonism between anti-apoptotic and pro-apoptotic members of Bcl-2 family, in which Bax inhibits the function of Bcl-2/Bcl-XL through protein–protein interaction [90]. Pim-1 kinase can directly phosphorylate Bad, a pro-apoptotic member of the BH3 family of proteins, and then weaken its binding ability with Bcl-2/Bcl-XL, thus re-activating these anti-apoptotic proteins [91].

Cyclin D1 (CCND1) is an essential modulator of G1/S phase transition of the cell cycle. *CCND1* overexpression is observed in many kinds of cancer. In this situation, G1/S phase transition of the cell cycle can be accelerated, therefore leading to uncontrolled cell proliferation.

Angiogenesis is an important physiological step to promote late tumor growth [92]. Tumors need a dedicated blood supply to provide the oxygen and other essential nutrients in order to grow beyond a certain size. When tumors grow to a certain extent, oxygen supply is far from enough to meet their needs. As a result, angiogenesis is eventually induced to further provide alternate routes of blood supply for the tumor. Moreover, neovascularization makes cancer cell metastasis more feasible since it provides a migration pathway due to the high permeability of the new vessel [93]. Therefore, angiogenesis may serve as a target for the treatment of various types of cancer.

#### Suppression of cancer cell proliferation and the promotion of apoptosis

**Fig. 2:**
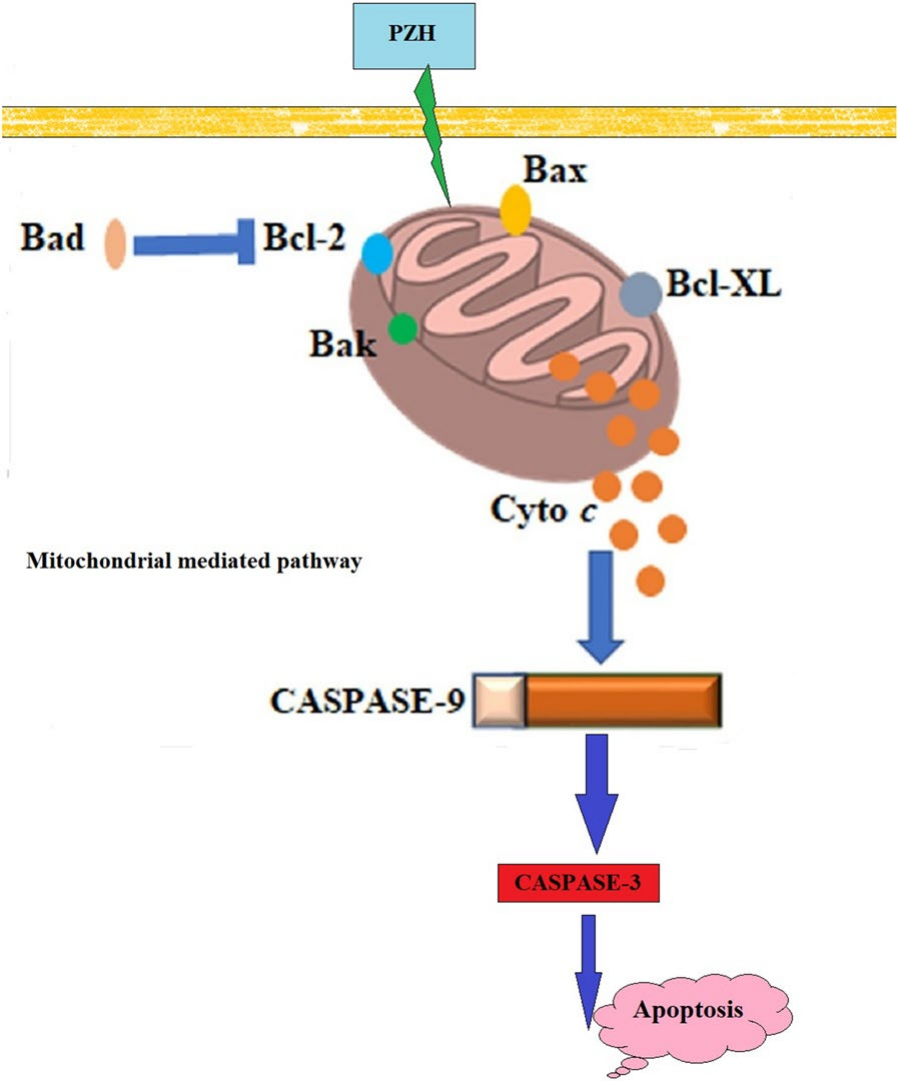
Schematic diagram on the effects of PZH on induction of apoptosis

PZH can potentially inhibit tumor growth in vivo and in vitro through different MOAs, by modulating multiple signaling pathways (Fig. 1). PZH treatment may inhibit tumor growth by suppressing cancer cell proliferation and/or promoting apoptosis [94–96]. Treatment with different doses of PZH can significantly suppress tumor growth of human malignant tumors transplanted into BALB/C nude mice [94]. The results have indicatedthat PZH can reduce the viability and induce apoptosis of HT-29 CRC cells [94]. PZH treatment has reportedly resulted in the collapse of the mitochondrial membrane potential, activation of caspase 3 and an increase in Bax/Bcl-2 ratios, suggesting an promotion of cancer cell apoptosis via regulation of Bcl-2 family and activation of caspase 3. Figure 2 represents a schematic diagram on the effects of PZH on induction of apoptosis. Interestingly, the rate of tumor growth inhibition improved after co-treatment with human P27KIP1 expressing AAV virus and PZH (from 34.1 to 63.8%), suggesting a synergistic effect and a potential molecular mechanism involving p27-related signaling pathways [97]. The inhibition of cell proliferation and promotion of apoptosis mediated by PZH have also been confirmed using a CRC mouse model [94]. It was shown that PZH’s anti-cancer activities might be related to the suppression of STAT3 signaling pathways, therefore resulting in the upregulation of Bax/Bcl-2 ratios as well as down-regulation of *CCND1* and *CDK4* expression. Overall, these effects can actually lead to the induction of apoptosis as well as the inhibition of cell proliferation. This also suggests that the suppression of STAT3 pathway might be one of the MOAs by which PZH can be used for CRC treatment. Moreover, PZH has been found to block the G1/S cell cycle progression and to suppress both mRNA and protein levels of CCND1 and CDK4 in Caco-2 CRC cells, suggesting that inhibition of cell proliferation via cell cycle arrest is a potential MOA through which PZH exerts its anti-cancer effecs. In addition, it has been reported that PZH treatment can largely inhibit IL-6-induced upregulation of Cyclin D1 and Bcl-2 (two main target genes of the STAT3 pathway) as well as increase the expression of SOCS3, indicating again that PZH may inhibit proliferation and promote apoptosis of human colon carcinoma cells via modulation of the IL-6/STAT3 signaling cascade [98]. Another study has shown that PZH may also suppress the proliferation of CRC cells by upregulating the expression of miR-34c-5p, providing a novel perspective for understanding the MOA of PZH, in the context of miRNA biology [99].

Further in vivo studies have indicated PZH can negatively modulate (i) 5-FU-induced diarrhea, (ii) apoptosis of intestinal crypt cells, and (iii) intestinal histopathological changes in CT-26 tumor-bearing mice. These biological effects are probably correlated with reduced levels of Baxand enhanced expression of anti-apoptotic Bcl-2 in the intestinal crypts [100]. These studies provide preliminary experimental data and theoretical basis for the clinical use of PZH in CRC.

#### *Suppression of tumor growth *via* the inhibition of tumor angiogenesis*

PZH treatment can significantly suppress tumor growth via the inhibition of both tumor angiogenesis [96, 98, 101] and lymphangiogenesis [41]. PZH is able to inhibit CRC growth by inhibiting tumor angiogenesis. In fact, PZH can reduce the intratumoral microvessel density (MVD) and the percentage of CD31 positive cells [101]. High expression of CD31 is correlated with a poor prognosis in CRC patients and, thus, it could be used to reflect the disease progress and/or as a prognosis indicator of CRC [102]. Using chick as a model, PZH has been shown to diminish the growth of blood vessels during embryogenesis and angiogenesis tube formation, as well as to decrease the proliferation and migration of Human Umbilical Vein Endothelial Cells (HUVECs) in a dose and time-dependent manner [96]. Moreover, PZH treatment may down-regulate both mRNA and protein levels of VEGF-A,VEGFR2, bFGF and bFGFR in both HUVECs and HT-29 CRC cells [101]. These effects are possibly related to the reduction on the activated status (i.e. phosporylated levels) of signaling enzymes and transcription factors including STAT3, ERK, Akt, JNK and p38 [103]. Importantly, PZH treatment can negatively affect the metastatic process of CRC cells via suppression of TGF-β signaling [104, 105]. In other word, PZH may inhibit metastasis of CRC cells by modulating TGF-β1/ZEB/miR-200 signaling network, particularly inhibiting the expression of key mediators of TGF-β1 signaling. As a result, PZH can further lead to a decrease in the cellular levels of N-cadherin (mesenchymal marker) and, concomitantly, an increase in the levels of E-cadherin (epithelial marker) [104]. Furthermore, PZH treatment can upregulate the expression of certain miRNAs, including miR-200a, miR-200b and miR-200c. Importantly, it has been reported that microRNA-200b and microRNA-200c promote CRC cell proliferation [106]. Moreover, PZH may suppress lymphangiogenesis via the downregulation of VEGF-C, a key regulator in cancer metastasis. This suggests that VEGF-C may act as a novel target of PZH for anti-lymphangiogenic therapy [41]. These results suggest that inhibiting tumor angiogenesis is one of the MOAs by which PZH may affect cancer progression.

#### Regulation through a complex signaling pathway networks

The PZH-mediated suppression of tumor growth in CRC, was independently confirmed in osteosarcoma [107] and ovarian cancer [108]. Recent studies have also indicated that PZH may significantly inhibit the growth of CRC stem cells via Notch signaling [109, 110]. More importantly, PZH treatment may overcome drug resistance in both CRC and osteosarcoma [111, 112]. For this, PZH may elevate the activity of caspase-3, caspase-9 and Bax, while decreasing the phosphorylation levels of Akt and PI3K in a dose-dependent manner. This series of events suggest that PZH may also exert its putative antitumoral activity by affecting PI3K/Akt signaling cascade [71]. Recently, it has been shown that PZH can additionally inhibit cancer cell proliferation and induce apoptosis in human hepatocellular carcinoma BEL-7402 HCC cells via upregulation of the tumor suppressor miR-16 [113].

#### The mechanisms of anti-drug resistance

So far, drug resistance is still a bottleneck in the treatment of various types of cancers.The putative effect of PZH on drug resistance and its underlying MOAs have been investigated lately. Indeed, it has been shown that PZH may play this role by inhibiting drug efflux and *ABCG2* expression, leading to a reduction onmulti-drug resistance (MDR)-induced EMT, suppression ofcancer cell migration/invasion and decreased activation of TGF- β signaling in vitro [111]. For instance, PZH appears to inhibit the proliferation of U2OS/ADM human osteosarcoma cells via G2/M cell cycle arrest and enhanced apoptosis due to the downregulation of Bcl-2, survivin and P-glycoprotein (P-gp) expression and upregulation of Bax. These findings clearly suggest that PZH can be a potential therapeutic agent against multidrug-resistant osteosarcoma, and warrants further investigations in vivo [112]. In addition, PZH treatment can upregulate the expression of miR-22, a tumor supressor miRNA, and downregulate the expression of its target gene *c-MYC*, suggesting that PZH could also overcome chemo-resistance in cancer cells by increasing miR-22 expression and, possibly, by reversing the imbalance between proliferation and apoptosis [71]. Furthermore, it has been demonstrated that PZH may also inhibit the viability of an ADR resistant human breast cancer cell line (MCF-7/ADR cells) in a dose-dependent manner [114]. Further analyses have shown an increased intercellular accumulation of ADR and a down-regulated expression of ABCG2 and ABCB1, which subsequently might induce protective effects in cells against chemotherapy-induced damage through increasing the doxorubicin efflux [114].

#### Anti-metastasis and the regulation through EMT

It has been reported that PZH could inhibit hypoxia-induced EMT, hypoxia-enhanced migration and invasion, and activation of HIF-1α pathway in colon carcinoma cells [115, 116]. PZH can also inhibit hypoxia-induced migration and tube formation and expression of HIF-1 and VEGF-A in HUVECs [115].

PZH has been shown to inhibit tumor liver metastasis, EMT and activation of TGF-β pathway in vivo in an orthotopic CRC mouse model [105]. Similarly, in vitro studies have shown that PZH can inhibit the proliferation, invasion and migration of OVCAR-3 cells, without any apparent apoptotic effect. Moreover, PZH can regulate the levels of signaling proteins involved in late cancer progression, including basal and activated forms (phosphorylated) of AKT and mTOR, as well as cell cyle modulators (CDK4, CDK6), tumor supressor genes and proto-oncogenes (p53 and c-Myc, respectively).

#### Regulation through stem-like side population (SP) cells

SP cells are similar to normal stem cells, since they are suitable to self-renewal and differentiation to mature somatic cells [117]. SP cells seem to play a critical role in the tumorigenesis. PZH has been reported to possibly reduce the percentage of SP in HT-29 cells [109]. Therefore, PZH treatment can significantly reduce the percentage of SP cells in a dose-dependent manner. In addition, PZH can inhibit, at higher extent, the viability and promot the apoptosis and differentiation of isolated SW480 SP cells, a subgroup of CRC with biological properties of tumor stem cells. Moreover, PZH profoundly (and negatively) affects the mRNA andprotein levels of Notch1 and Hes1 in SP cells [110]. Notch1 and its target Hes1 play a key role in the regulation of tumor cell proliferation and induction of apoptosis. These findings suggest that PZH can negatively modulate the characteristics of CSCs by suppressing Notch1 signaling.

### Anti-inflammatory effects

The pharmacological effects of PZH on altering the expression of the IL-6 and STAT3 suggest its therapeutic potentials on the treatment of inflammatory diseases, such as ulcerative colitis (UC). Actually, administration of PZH has potentially prevented DSS-induced colon shortening, as well as ameliorating colonic histopathological changes (such as mucosal ulceration), infiltration of inflammatory cells, crypt distortion and diminishing hyperplastic epithelium. Moreover, PZH can markedly inhibit the serum levels of the inflammatory biomarker serum amylase A (SAA) in UC mice, probably through inhibiting IL-6/STAT3 cascades [118].

## Conclusion

Exploring the mechanistic basis of PZH on chronic inflammation and immune regulation is essential to consolidate pharmaceutical evidence for its clinical use as an immunomodulator in high impact conditions such as cancer. Further clinical studies focusing on PZH for the treatment of NASH, liver fibrosis and alcoholic liver diseases will provide evidence-based validation of its clinical efficacy and potential advantages. Actually, the potential use of PZH as an adjuvant with other first-line therapies may also be of great significance for the therapy of major chronic disorders.

## Data Availability

The data used to support the findings of this study are available from the author upon request.

## Abbreviations

ALD: Alcoholic liver disease
Bax: BCL2-associated X
Bcl-xL: B-cell leukemia/lymphoma 2 xL
Beta-ODAP: Beta-*N*-oxalyl-l-alpha,beta-diaminopropionic acid
CCl4: Carbon tetrachloride
CNS: Central nervous system
CRC: Colorectal cancer
CCND1: Cyclin D1
CNKI: China National Knowledge Infrastructure
EAE: Experimental autoimmune encephalomyelitis
ER: Endoplasmic reticulum
ERS: Endoplasmic reticulum stress
FXR: Farnesoid X receptor
HUVECs: Human Umbilical Vein Endothelial Cells
HFD: High fat diet
HPLC: High performance liquid chromatography
Hcy: Homocysteine
LPS: Lipopolysaccharide
MDR: Multi-drug resistance
MOA: Mechanism of action
MVD: Microvessel density
MS: Multiple sclerosis
NAFLD: Non-alcoholic fatty liver disease
NF-κB: Nucleartranscription factor-κB
PZH: Pien Tze Huang
P-gp: P-glycoprotein
RER: Rough ER
SAA: Serum amylase A
SHP: Small heterodimer partner
SP: Side population
SREBP1c: Sterol-regulatory element binding protein-1c
TCM: Traditional Chinese Medicine
UC: Ulcerative colitis
STAT3: Signal transducer andactivator of transcription 3
5-FU: 5-Fluorouracil
